# Herbst’s New Method of Filling Teeth

**Published:** 1884-10

**Authors:** W. D. Miller

**Affiliations:** Berlin, Germany


### Independent Practitioner.

Vol. V. October, 1884. No. 10.

### Original Communications.

### HERBST'S NEW METHOD OF FILLING TEETH.

BY DR. W. D. MILLER, BERLIN, GERMANY.

The method of inserting gold fillings strenuously advocated by W. Herbst, of Bremen, during the last half decade, is sufficiently well known to the profession to render a detailed description unnecessary. This method is especially characterized by two features. The first of these is the extensive use of the matrix for approximal cavities, the second the employment of a rotating, smooth-pointed instrument for condensing the gold.

The operation may be best made clear by describing a case in practice. We have to fill the left upper bicuspids, both decayed on the approximal surfaces. The cavities are opened to the grinding surface and prepared as for inserting a cohesive foil filling, with exception of retaining points, which are never made. The matrix is now brought into place, and the point of a steel pin inserted between the matrix and first bicuspid from the buccal side, and if necessary from the lingual side also, so as to press the matrix tight against the surface of the second bicuspid at the neck; warmed shellac is now packed into the cavity of the first bicuspid, to hold the matrix firmly in position. After the cavity of the second bicuspid has been filled, the shellac and matrix are removed, a second higher (wider) matrix inserted and pressed by the same means as before, tight against the surface of the first bicuspid, which is then ready for filling. Should the second bicuspid be wanting, then the matrix is held in position by filling the whole space between the first bicuspid and the first molar with shellac. The matrix being in position, three or four cylinders of Wolrab foil No. 2, 3 or 4 (or an equivalent of Wolrab foil No. 4 made into pellets) are brought into the cavity just as they would be into a simple cavity on the grinding surface, and fixed into position by pressing tightly upon them with one of the larger burnishers of the set, slowly rotating. It is then condensed by a succession of strong taps or pressures of the rapidly rotating instrument No. 5.

(The surface of the instrument soon becomes coated with gold, which must be removed by pressing it while rotating upon a piece of pure tin.)

This is then followed up by an ordinary hand plugger, to ascertain whether the gold has been properly condensed at all points. The above process is repeated till the operation is completed, the surface of the gold at last being thoroughly condensed by means of one of the smooth polished instruments of the set.

In filling large cavities on the grinding surface, first two, three, or four cylinders of No. 1, 2 or 3 Wolrab foil are pressed against the bottom of the cavity with the pliers, and then with one of the *polished* instruments, *slowly* rotating, fixed in position; if it does not at once become firm, more gold is put in till it does; it is then condensed with No. 5, *rapidly* rotating, and this instrument is then used almost exclusively till the gold is all in; then No. 1–4 or 7 is used for condensing the surface.

Approximal cavities in the central incisors are treated as follows (previous separation by means of cotton having been secured): The cavities are prepared slightly broader at the bottom than at the opening; no retaining points are required. Two or three cylinders of No. 2 Wolrab foil are pressed into the first tooth towards the gums, likewise into the second tooth, and condensed with No. 5; this process being repeated till both cavities are overfilled, so that the gold in the two fillings appears to form one continuous mass.

Then No. 15 (a clean, fine needle), slowly rotating, is pressed through between the filling at different points, and the separation completed with a fine file. The fillings are then further condensed by Nos. 12–14, rapidly rotating, with interrupted pressure.

The advantages claimed for the Herbst method are: 1st. Rapidity of operation. This comes particularly into account in proximal fillings, which can be inserted in much less time than is required by either the hand or electric mallet.

2d. The injuries frequently done to frail tooth structure, as well as to the pericementum by the use of heavy mallets, of course are entirely avoided.

3d. The equally baneful necessity of weakening or sapping a tooth by large retaining points is also done away with.

4th. The operation is less disagreeable to the patient than that by the mallet method.

Perhaps the most valuable feature of any method is the ability to produce by it fillings which shall be perfectly adapted to the walls of the cavity, and in this respect the Herbst method seems to promise success.

A number of fillings made by Herbst, with the time occupied in the insertion of each, were sent to me with the request that I would give them a thorough examination. For this purpose I caused similar fillings to be inserted in the same time by the hand mallet and by Telchow's Pneumatic Mallet. The teeth were then immersed in an alcoholic solution of fuchsine and allowed to remain twenty-four hours; at the end of this time they were split, and it was found that Herbst's fillings had resisted the penetrating power of the liquid better than the others; *i. e.*, they made better contact with the walls of the cavity than those inserted by the other methods. Herbst makes use of gold cylinders prepared by Wolrab in Bremen, a preparation which works, in the words of an old operator, "like mush."

How far his success is to be ascribed to the admirable qualities of the gold I do not know, nor is it possible to say how his fillings will stand the test of time.

This much, however, we may safely say, that his method has shown itself to be deserving of a thorough investigation, and it is particularly desirable that American operators should make themselves acquainted with it.

Cuts of the instruments employed by Herbst may be found in the number for February, 1884.—EDITOR.

